# Structure-Properties Correlations of PVA-Cellulose Based Nanocomposite Films for Food Packaging Applications

**DOI:** 10.3390/polym17141911

**Published:** 2025-07-10

**Authors:** Konstantinos Papapetros, Georgios N. Mathioudakis, Dionysios Vroulias, Nikolaos Koutroumanis, George A. Voyiatzis, Konstantinos S. Andrikopoulos

**Affiliations:** 1 Foundation for Research and Technology—Hellas (FORTH), Institute of Chemical Engineering Science (ICE-HT), Stadiou Street, 265 04 Patras, Greece; kpapapetros@iceht.forth.gr (K.P.); mathioy@iceht.forth.gr (G.N.M.); dvroulias@iceht.forth.gr (D.V.); 2 Department of Chemical Engineering, University of Patras, 265 04 Patras, Greece; 3 Application Driven Research & Innovative Engineering (ADRINE), Patras Science Park, Stadiou Street, Platani, 265 04 Patras, Greece; nkoutrou@adrine.gr; 4 Department of Physics, University of Patras, 265 04 Patras, Greece

**Keywords:** PVA, nanocellulose, lignocellulose, nanocomposites, active food packaging

## Abstract

Bio-nanocomposites based on poly (vinyl alcohol) (PVA) and cellulosic nanostructures are favorable for active food packaging applications. The current study systematically investigates the mechanical properties, gas permeation, and swelling parameters of PVA composites with cellulose nanocrystals (CNC) or nano lignocellulose (NLC) fibers. Alterations in these macroscopic properties, which are critical for food packaging applications, are correlated with structural information at the molecular level. Strong interactions between the fillers and polymer host matrix were observed, while the PVA crystallinity exhibited a maximum at ~1% loading. Finally, the orientation of the PVA nanocrystals in the uniaxially stretched samples was found to depend non-monotonically on the CNC loading and draw ratio. Concerning the macroscopic properties of the composites, the swelling properties were reduced for the D1 food simulant, while for water, a considerable decrease was observed only when high NLC loadings were involved. Furthermore, although the water vapor transmission rates are roughly similar for all samples, the CO_2_, N_2_, and O_2_ gas permeabilities are low, exhibiting further decrease in the 1% and 1–5% loading for CNC and NLC composites, respectively. The mechanical properties were considerably altered as a consequence of the good dispersion of the filler, increased crystallinity of the polymer matrix, and morphology of the filler. Thus, up to ~50%/~170% enhancement of the Young’s modulus and up to ~20%/~50% enhancement of the tensile strength are observed for the CNC/NLC composites. Interestingly, the elongation at break is also increased by ~20% for CNC composites, while it is reduced by ~40% for the NLC composites, signifying the favorable/unfavorable interactions of cellulose/lignin with the matrix.

## 1. Introduction

In recent years, there has been a growing interest in developing sustainable, high-performance materials for food packaging. Several material properties must be taken into account, including barrier properties (especially referring to gases such as O_2_ water vapors and CO_2_), mechanical properties (defined with respect to the specific packaging product), chemical resistance properties (for example, maintaining properties under weak/strong acidic solution environments), chemistry of degradation and biodegradability, antimicrobial properties, and compliance with international/local food regulations [1,2]. The properties of materials related to gas permeability are crucial for extending the shelf life of perishable foods via modified atmosphere packaging. On one hand, there is a necessity for new materials with enhanced barrier properties, and on the other hand, there are requirements associated with the optimization of the current material properties.

The hydroxyl groups in poly (Vinyl Alcohol) (PVA) contribute to strong hydrogen bonding both intra- and inter-molecular, leading to a particularly attractive polymer matrix due to its excellent film-forming properties, biodegradability, and inherent barrier performance [3]. Due to its hydrophilic nature and water-swelling properties, PVA is preferentially used for hydrogel production in biomedical and other applications by crosslinking PVA solutions with various chemicals (e.g., glutaraldehyde and sodium nitrate) or physical treatments (e.g., freeze–thaw) [4,5,6]. Enhancing the matrix characteristics using these approaches often requires additional processing steps, increased energy consumption, or potentially harmful chemicals. To eliminate these additional requirements and consequences, the development of composite materials seems to be the most beneficial strategy for preserving or even improving the inherent properties of the polymer, while introducing new functionalities in several cases [7]. Moreover, composites can often be processed using conventional methods, leading to cost-effective and environmentally friendly manufacturing [8,9,10].

One effective strategy for both enhancing the overall properties and eco-friendly profile of PVA is to develop composites that incorporate inexpensive, biodegradable, and easily processable cellulose-based fillers. PVA is particularly compatible with cellulosic materials since both are highly polar. Moreover, nanocelluloses are increasingly favored in applications such as food packaging, biosensors, biomedicine, and food additives because of their intrinsic properties, including biodegradability and stability in aqueous environments, and they are non-toxic [11]. Several studies have reported PVA-based nanocomposites with cellulose nano crystals (CNCs) to enhance various properties, such as mechanical, thermal, and swelling properties [12,13,14,15,16,17]. CNCs are produced after acid hydrolysis of cellulose-based materials, forming rod-like nanoparticles, which are known for their incorporation as fillers in several polymeric matrices, aiming in most cases at the enhanced mechanical properties of the composites. Conversely, fewer studies have focused on ligno-cellulosic composites, although the number of studies has significantly increased in recent years [18,19]. Although most PVA/cellulose-based composites have shown a significant improvement in mechanical and barrier properties, no systematic correlation of physicochemical properties with the type, morphology, and concentration of cellulosic inclusions has been attained, and conclusive arguments relating the structure at the molecular level and the macroscopic properties of the composites have not yet been accomplished [12,13,14,15,16,17].

The current work focuses on a systematic parallel study of two types of PVA composites containing cellulose-based fillers: CNC with a relatively low aspect ratio or a fiber-like mildly treated lignocellulose (NLC). A series of experiments enabled a detailed structural study of the composites and the evaluation of their respective macroscopic properties, which are critical for food packaging applications. To this end, the morphology and structure at the molecular level of both the filler and host polymer were thoroughly studied. Hydrogen binding between the two components is highlighted, and the crystallinity is revealed as a function of composition. Furthermore, polarized Raman spectroscopy was applied in order to semi-quantitatively characterize the tendency of the host polymer nanocrystals to orient under uniaxial deformation. Interesting results were obtained since the degree of their orientation was found to depend not only on the loading but also on the final draw ratio. Understanding and controlling these parameters are of primary importance for designing durable packaging that exhibits mechanical anisotropy. In contrast, macroscopically observed properties, which are of primary importance for food packaging applications, such as swelling, mechanical, and barrier properties, were systematically measured, and insights into structure-property correlations were exploited to ensure that the properties of the composite material surpass those of the neat PVA polymer. Our findings enable the selection of appropriate compositions that tailor the macroscopic properties to meet the requirements of specific food packaging applications.

## 2. Materials and Methods

### 2.1. Materials

Polyvinyl Alcohol (PVA) (Mw of 89–98 kDa, 99+% hydrolyzed, CAS Number: 9002-89-5) was purchased from Sigma-Aldrich (Steinheim, Germany). Aqueous 11.5–12.5 wt.% Cellulose NanoCrystals suspension (CAS Number: 9004-34-6) was purchased from the University of Maine (Orono, ME, USA). Lignocellulose from hardwood (after ball milling, 4.56 wt.% suspension) was kindly granted by the Laboratory of Polymer Reaction Engineering, CERTH (Thessaloniki, Greece). Ultrapure water was obtained using a Milli-Q RG (MilliporeSigma, Darmstadt, Germany) apparatus water purification unit.

### 2.2. Sample Preparation

Aqueous solutions of 4 wt.% PVA were prepared by dissolving the polymer in triple-distilled water at 85 °C under stirring for 1 h. After stirring, the transparent PVA solution was allowed to cool to room temperature. Cellulose-based composites were formed by dispersing appropriate amounts of CNC or lignocellulose in aqueous solutions of PVA. The nanocellulose materials were used in the form of slurry (viscous water dispersions) to be stored for longer periods without aggregation. The slurry was diluted in triple-distilled water before mixing with appropriate amounts of the PVA solution. The final suspension was sonicated for 10 min at 45 W. PVA solutions and the respective nanocellulose suspensions were cast on glass Petri dishes and dried for 1 week at ambient room temperature. The resulting cast films were further dried for 4 h in a vacuum oven at 40 °C before characterization (Figure S1). Pristine PVA films were produced, as well as their composites, exhibiting 1, 5, and 10 wt. % of cellulosic inclusions (either CNC or lignocellulose). The selected wt.% covers the low-moderate range as higher loadings result in agglomeration, which deteriorates the composites’ properties. Replicate samples for each composition were prepared for statistical analysis. For the FTIR experiments, five replicates were measured; three replicate samples were used for the XRD, DSC, gas permeability measurements, and the extraction of swelling properties, while the wet cup method was applied to two replicate samples for each composition. The average and standard deviation of the measurements of the available replicate samples were calculated for each composition. A summary of the samples prepared, along with the experimental techniques used for their characterization, is presented in Table 1.

### 2.3. Analytical Techniques

#### 2.3.1. Scanning Electron Microscopy (SEM)

Scanning Electron Microscopy (SEM) was used to assess the morphological features and dimensions of the cellulosic inclusions, as well as their dispersion within the PVA matrix. To detect notable variations in potential defects and agglomerations by increasing the filler content, a nitrogen cryo-cut of the films was deployed. The images were collected using a Zeiss SUPRA 35VP Scanning Electron Microscope (Carl Zeiss Microscopy GmbH, Jena, Germany) system operating at 2–10 kV voltage, depending on the sensitivity of each sample (2 kV for film cross-sections and 5–10 kV for cellulosic inclusions). This characterization process provided an in-depth analysis of the microstructural features of the material and filler distribution, which are essential for assessing the effectiveness of the incorporation strategy.

#### 2.3.2. Attenuated Total Reflection Fourier Transform Infrared Spectroscopy (ATR-FTIR)

The ATR-FTIR spectra of the solid samples were recorded on an Alpha-II Diamond ATR Spectrometer (Bruker Optics GmbH, Ettlingen, Germany) in the range of 400–4000 cm^−1^. The spectral resolution was set to 4 cm^−1^.

#### 2.3.3. X-Ray Diffraction (XRD)

X-ray diffraction was used to identify the crystalline phase, measure the crystallinity of the samples, and detect changes in the PVA crystal formation after the addition of various loadings of cellulosic materials. XRD measurements were carried out using a Bruker D8 Advance diffractometer (Bruker AXS GmbH, Karlsruhe, Germany) equipped with a Cu lamp (λCuKa = 1.54046 Å) at a scanning rate of 0.5°/min over a range of 5–30° (2θ). The crystallinity index (C.I.) of PVA in each case was calculated using the amorphous subtraction method. The latter was achieved by data fitting, where the raw XRD data were deconvoluted into individual crystalline peaks (of both PVA and cellulose contributions) and a broad amorphous halo. Once the amorphous halo was subtracted from the total diffraction pattern, the crystallinity index (Cr.I%) was calculated as follows:

(1) $$Cr.I(\%)=\frac{Icrystalline}{Itotal}\times 100$$

where *Icrystalline* is the integrated intensity of the fitted crystalline peaks, and *Itotal* represents the total scattered intensity before amorphous subtraction.

The crystallite size, *D*, refers to the average crystal width and can be calculated using Scherrer’s equation.

(2) $$D=\frac{K\lambda }{FWHM\;cos\theta }$$

where *K* is a dimensionless factor that depends on the crystal shape (*K* = 0.94 in this study, describing elongated crystals), *λ* is the wavelength of the incident X-ray radiation, *FWHM* is the full width at half maximum of the peak at *θ* (in radians), and *θ* is the angle of the diffraction peak of the crystalline phase (Bragg’s angle).

#### 2.3.4. Differential Scanning Calorimetry (DSC)

Differential scanning calorimetry (DSC) measurements were performed using a Q100 system (TA Instruments, New Castle, DE, USA) equipped with a liquid nitrogen cooling accessory. To investigate the melting behavior of the neat polymer and nanocomposites, approximately 6–8 mg of each sample was heated from room temperature to 240 °C at a rate of 10 °C min^−1^ under a nitrogen flow of 50 mL min^−1^. Data were collected from the first heating cycle to reflect the actual state of the prepared composites. The crystallinity index (*Cr.I*%) of the PVA composites was determined from the endothermic melt peaks using the following equation:

(3) $$Cr.I\;(\%)=\frac{{\Delta H}_{m}}{{\Delta H}_{m}^{0}(1-\varphi )}\times 100$$

where ${\Delta H}_{m}$ is the measured heat fusion, ${\Delta H}_{m}^{0}$ is the standard heat fusion for 100% crystalline PVA (138.6 J g^−1^) [20], and φ is the weight fraction of the fillers.

#### 2.3.5. Gas Permeability Measurements

Single gas permeation measurements were performed using the Wicke-Kallenbach method. A circular membrane film was placed inside a custom-built permeation cell with an effective membrane area of 6.97 cm^2^. The retentate and permeate compartments of the cell were sealed with Viton O-rings and secured using a vacuum clamp. To maintain a measurement temperature of 30 °C, two coil heaters (BL Sistemi S.R.L., Rome, Italy) equipped with a thermocouple (RS Components Ltd, Corby, UK) were connected to a temperature controller and integrated into the permeation cell. Gas flow was regulated using mass flow controllers (FC7700C, Aera, Hachioji, Japan), with atmospheric pressure applied to both the retentate and permeate sides. During the single gas permeability test, helium (used as a sweep gas) was introduced at a flow rate of 20 cm^3^ min^−1^. The gas composition on the permeate side was analyzed using a Shimadzu gas chromatograph (GC-2014) (Shimadzu Corporation, Kyoto, Japan) equipped with a thermal conductivity detector. Porapak Q (Waters Corporation, Milford, MA, USA) was employed for CO_2_ analysis in the helium permeate stream. The detection limit for CO_2_ was 10 ppm using a thermal conductivity detector. Considering the membrane thickness and effective area of the membrane, the CO_2_ permeability that can be detected is up to 0.01 Barrer. For N_2_ gas, the detection limit is 100 ppm using a thermal conductivity detector, which means that the respective permeability that can be detected is up to 0.1 Barrer. Therefore, N_2_ and O_2_ were analyzed using a quadrupole mass spectrometer equipped with a secondary electron multiplier detector. The detection limit for these gases is <1 ppm, with a sensitivity of 10 ppb. The *m*/*z* = 32 signal was calibrated by introducing a known quantity of O_2_.

#### 2.3.6. Water Vapor Transmittance Rate Measurements

The water vapor transmission rate (WVTR) of the composite membranes was measured using the “wet cup” method outlined in corresponding ASTM [21]. In ASTM terminology, WVTR is defined as the steady-state flow of water vapor per unit time through a unit area of the membrane, perpendicular to its surface, under specified temperature and humidity conditions on both sides. For this measurement, an acetal dish filled with distilled water was tightly sealed with the test membrane and placed in a custom-made chamber maintained at ~30 °C and 21% relative humidity (RH) [22]. The least-squares method was applied to the linear part of the mass change over time graph to determine the steady-state water vapor transmission rate. The WVTR was calculated from the steady-state region using equations reported elsewhere [22,23].

#### 2.3.7. Swelling Properties

Swelling tests were performed in three different media: distilled water, ethanol, and a 50% water/ethanol mixture at 25 °C. Prior to immersion, all samples were dried and then incubated at R.T. for up to 48 h. Each sample was removed from the container, excess water was removed by wiping with filter paper, and then it was weighed. The equilibrium swelling degree (*S*%) was calculated using the following equation:

(4) $$S\%=\frac{S_{f}-S_{i}}{S_{i}}\times 100$$

where $S_{i}$ is the initial weight of the sample before immersion, and $S_{f}$ is the weight at equilibrium water content.

#### 2.3.8. Mechanical Properties

A hydraulic mechanical testing system (MTS R58 Mini Bionix, MTS Systems Corporation, Eden Prairie, MN, USA) was employed for the tensile tests of the pure and composite films. The MTS system was equipped with a load cell of 25 kN. The tensile properties of the PVA composite films were determined according to the modified ASTM D882 [24]. The initial sample length was 3 cm, and the crosshead moving speed was set to 6 mm/min. According to ASTM D882, eight replicate experiments were performed. From the stress–strain curves, the Young’s modulus, tensile strength, and elongation at break were extracted.

#### 2.3.9. Raman Scattering

Backscattering Raman spectra were collected using the T-64000 model of Jobin Yvon (Horiba, Ltd., Kyoto, Japan) excited with a Cobolt Fandango TM ISO laser operating at 514.5 nm. The excitation beam was directed to the sample compartment of a metallurgical microscope (Olympus BHSM-BH2, Olympus Corporation, Tokyo, Japan). A microscope was used to deliver the excitation laser beam to the sample and collect the backscattered light through a beam splitter and an objective lens adapted to the microscope aperture. The focusing objective was a Long Working distance (10 mm) 50×/0.55 Olympus lens. The samples were excited using a 6.5 mW laser power (measured after the microscope objective) for a total collection time of 1500 s. The scattered radiation was focused on the slit of a single monochromator after passing through an appropriate edge filter for the elastic scattering rejection (LP02-514RU-25, Laser 2000, Cambridge, UK). The dispersion and detection of the Raman photons were performed using a 600-grooves/mm grating and a 2D CCD detector (operating at 140 K), respectively. The total response of the system and the polarization calibration were checked using CCl_4_ as a reference. The notation of Raman polarization measurements comprises a combination of three letters, e.g., v-VV. The lowercase letter (v) denotes the orientation of the draw axis, while the two capital letters (HH, VV) denote the polarization direction of the excitation and scattered light, respectively; H, when the polarization is perpendicular to the draw axis, and V, when the polarization is parallel to it.

## 3. Results and Discussion

### 3.1. Morphology of the Films

**Figure 1:**
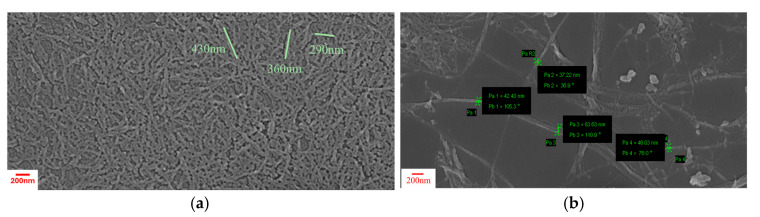
SEM images of nano-cellulosic inclusions. (**a**) CNC and (**b**) lignocellulose.

Figure 1 shows the SEM images of the cellulosic materials after drop casting onto a silicon wafer. Well-dispersed, needle-shaped structures of CNC were resolved, with a mean width of 40 ± 10 nm and a characteristic length (350 ± 100 nm), resulting in an aspect ratio of around 10 (Figure 1a). A high-magnification image of lignocellulose highlights the presence of a nanofibrous network, with a mean fiber diameter of 50 ± 20 nm and length of up to a few microns, leading to an aspect ratio of ~100 (Figure 1b).

**Figure 2:**
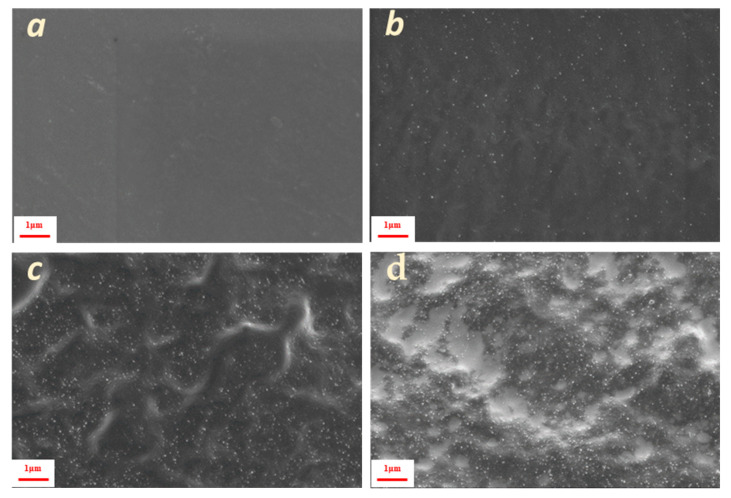
Cross-sectional surface SEM images of: (**a**) PVA pure, (**b**) 1%, (**c**) 5% and (**d**) 10% CNC composite.

SEM images of fractured surfaces of the composites revealed that pure PVA matrix exhibited a smooth morphology without cracks or voids (Figure 2a). In the PVA 1% CNC sample, white nanodots were observed on the cross-sectional surface (Figure 2b), suggesting good dispersion with limited cellulose aggregation in the PVA matrix. A similar good dispersion was also found for higher CNC contents (Figure 2c,d), resulting in highly transparent films, as seen macroscopically (Figure S1). Good CNC dispersion demonstrates the enhanced compatibility of the hydrophilic crystalline nanocellulose and PVA matrix and suggests considerable interaction between the filler and matrix. At higher cellulose loading values, crater-like areas were observed, probably due to the enhanced stiffness of the material [25].

**Figure 3:**
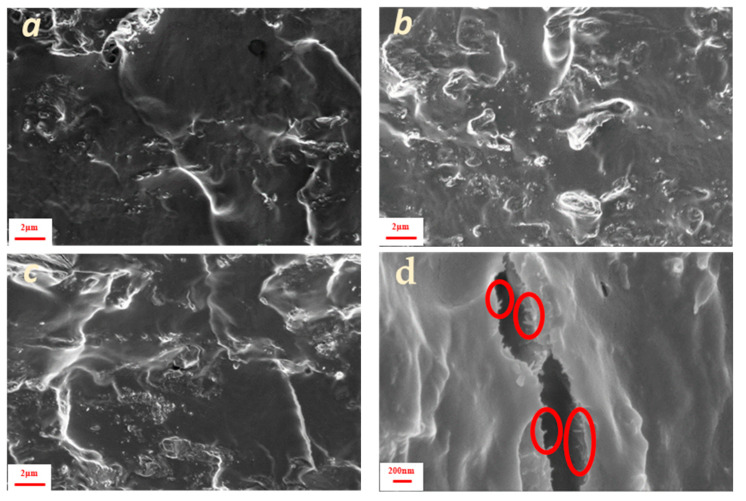
Cross-sectional surface SEM images of: (**a**) 1%, (**b**) 5%, and (**c**) 10% PVA lignocellulose composites. (**d**) 10% composite at a higher magnification; indicative lignocellulose nanofibers are encircled (red circles).

In contrast, the fibrous morphology of lignocellulose cannot be easily resolved in the cross-sections (Figure 3a). Only the terminal edges of the fibers protruded from the surface, while fibers parallel to the surface were detected only on small cracks, as shown in Figure 3d. As the percentage of inclusions increased, the roughness of the cryo-cut surface tended to increase, and the dispersion became less homogeneous (Figure 3b,c), which can also be verified macroscopically (Figure S1).

### 3.2. Structure at Molecular Level

#### 3.2.1. Conformational Alterations and H-Bonds of Cellulose in the Composites

FTIR spectroscopy is a sensitive technique for investigating the stereochemical and conformational structures of polymer chains, as well as for examining macromolecular interactions and their orientational and crystalline order [26]. Changes in the structure of cellulose could also be highlighted after possible interaction with PVA (through hydrogen bonding) or conformational changes [27].

**Figure 4:**
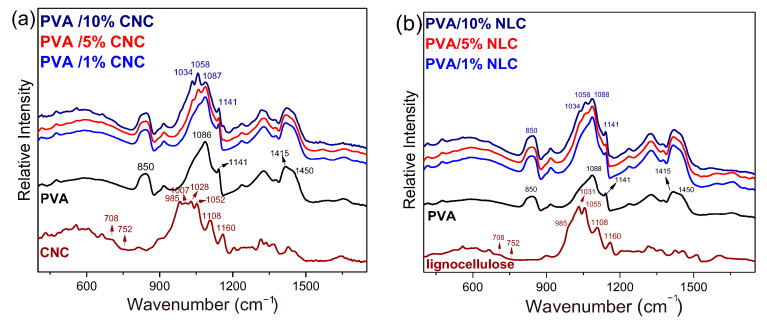
(**a**) ATR/FTIR spectra of CNC, neat PVA film, and PVA composites with 1, 5, and 10 wt.% CNC % in CNC. (**b**) ATR/FTIR spectra of NLC, neat PVA film, and PVA composites with 1, 5, and 10 wt.% in lignocellulose.

Therefore, to confirm the presence of nanocelluloses in the PVA matrix and observe any possible interactions between them, ATR/FTIR analysis was performed, and the results are depicted in Figure 4. The spectrum of the pure PVA film exhibits several intense peaks in the high-frequency spectral region, as well as CH_2_ bending at 1450 and 1415 cm^−1^ and C-OH stretching vibrations in the 950–1150 cm^−1^ spectral range. The characteristic bands weaker in intensity are the crystalline sensitive O-C-C bond stretching vibration at 1141 cm^−1^ [26] and the bands in the range of 1700 to 1550 cm^−1^, which are ascribed to the bending vibrations of water molecules adsorbed in the polymer matrix, although minor contributions from carbonyl groups (C=O and C=C stretching) may also appear due to residual acetate groups [17]. Finally, bands in the 850 cm^−1^ region are generally associated with C-C backbone stretching and CH_2_ rocking vibrations from the methylene groups in the polymer backbone [28,29,30].

Similar to PVA, cellulose exhibits strong vibrational bands in the 1070–950 cm^−1^ spectral range, which are typically assigned to vibrations involving C-O stretching within the glucopyranose units. In their detailed vibrational study of Iβ cellulose crystals under temperature and humidity-controlled experiments, Maréchal and Chanzy [27] attributed several modes of this particular spectral range to C-O vibrations of the primary (C6H_2_-O6H, bands at ~1000, 1015, 1035 cm^−1^) and the two secondary alcohols (more specifically C3-O3H, at ~1060 cm^−1^ and C2-O2H at ~1110 cm^−1^) of each glucopyranose unit. The three distinct peaks attributed to the primary alcohols corresponded to three respective conformations defined after rotation of the C6H_2_-O6H group with respect to the C5–C6 bond (the dominant one being that at ~1035 cm^−1^ for the Iβ crystal).

The vibrational energy of primarily the O-H modes, as well as the adjacent C-O modes described above, is strongly affected by intra- and inter-molecular hydrogen bonds. Differentiation in the strength of these hydrogen bonds due to physical and/or chemical transformations may result in peak shifts, which may be as high as ~100 cm^−1^ for the O-H modes and on the order of ~10 cm^−1^ for the C-O modes [31]. It has already been discussed [27] that an increase in the hydrogen bond strength lowers the O-H frequency and has the opposite effect on the associated C-O frequency. The characteristic cellulose C-O vibrations of both primary and secondary alcohols in the CNC, observed at 1028 and 1052 cm^−1^, respectively, exhibited a 6 cm^−1^ blue shift in the composite spectra (Figure 4a, observed at 1034 and 1058 cm^−1^). The blue shift of the CNC C-O frequencies experimentally detected in their composites suggests that the corresponding primary and secondary alcohols experience stronger hydrogen bonds with the polymeric matrix. These interactions may also lead to conformational alterations of the cellulose chains, explaining the considerable intensity decrease of the 985 and 1007 cm^−1^ bands in the composite spectra.

Regarding the molecular level structure of cellulose in the lignocellulose-PVA system, an analogous discussion can be made for CNC and its composites. The intensity contribution to the vibrational peaks attributed to the three different chain conformations of the primary alcohols was different for the lignocellulose sample compared to that of the CNC sample. Furthermore, the frequencies of the characteristic C-O vibrational modes of primary and secondary alcohols are slightly higher than those of CNC (1031 and 1055 cm^−1^), which shift even more when lignocellulose is incorporated into PVA (Figure 4b).

#### 3.2.2. Study of the Crystalline Phases

**Figure 5:**
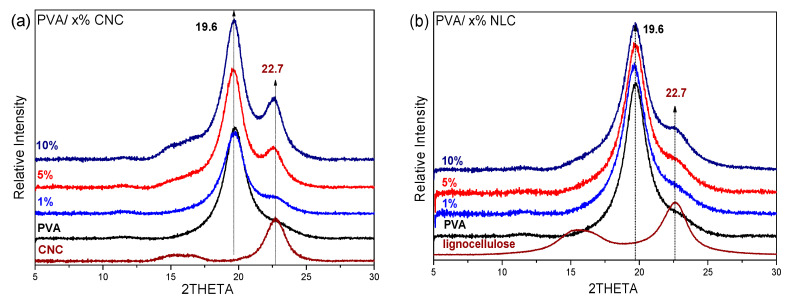
(**a**) XRD graph of Pure PVA film, cellulosic inclusion, and PVA composites: 1%, 5%, and 10% CNC. (**b**) XRD patterns of Pure PVA film, lignocellulose inclusion, and PVA composites with 1%, 5%, and 10% lignocellulose.

The dominant crystal lattice of the cellulose nanocrystals was Iβ, as indicated by both the FTIR and XRD data. More specifically, the typical band at 710 cm^−1^ attributed to the monoclinic, Iβ, crystal is evident in the FTIR spectra of CNC and lignocellulose (Figure 4), while the one at ~750 cm^−1^ associated with the triclinic, Iα, crystal is hardly resolved. In addition, both reference CNC (Figure 5a) and NLC (Figure 5b) exhibit a characteristic peak at 22.7° (200) along with a doublet at 15° (1$\bar{1}$0), and 16.5° (110), in their XRD diffractograms, all assigned to the crystal lattice of cellulose Iβ [32,33]. The monoclinic unit cell is characteristic of the polymeric PVA matrix, indicated by the strong peak at 2θ = 19.8° (10$\bar{1}$) and the weak shoulder at 22.8° (101) [34]. The crystallinity index (Cr.I%) can be estimated from FTIR spectra using several methodologies [24,33]. Several studies focused on the intensity alterations of the 1141 cm^−1^ band relative to the reference. A general linear equation was used to estimate the crystallinity index:

(5) $$Cr.I\;(\%)=A\left(\frac{a}{b}\right)+B$$

where *a* and *b* are the intensities of the 1141 cm^−1^ peak and the reference peak, respectively, and *A* and *B* are constants obtained by the cross-examination of additional experimental techniques, such as XRD or DSC. Mallapragada & Peppas (1996) used the distinct CH_2_ bend at 850 cm^−1^ as a reference band and calculated the constants A, B in Equation (5), using DSC crystallinity indexes [35]. Tretinnikov et al. (2012) used the adjacent 1088 cm^−1^ C-O stretching vibration as reference, while A and B constants were found through XRD experiments [26]. We calculated the Cr.I% following the work of Tretinnikov et al. [26], using the corresponding values of A and B as 89.5 and −13.1. A maximum Cr.I (%) of PVA was observed at 1% loading for both CNC and NLC (Table 2).

In addition to the reference materials, Figure 5 includes the XRD data of the PVA/x% CNC and PVA/x% lignocellulose composites. With respect to cellulosic materials, CNC is more crystalline as a consequence of the presence of amorphous residual plant parts in the lignocellulosic fibers [36], with the extracted crystallinity being around 85% for lignocellulose and 95% for CNC.

As illustrated in Figure 5, the diffractograms for the PVA/CNC (left) and PVA/lignocellulose composites (right) show diffraction peaks at 19.6° and 22.7°, corresponding to the PVA matrix and cellulosic inclusions, respectively. This indicates that the crystalline structure of PVA was largely maintained after the incorporation of either CNC or lignocellulose. However, the crystallinity of PVA, calculated using Equation (1) (example of deconvolution process found in Figure S2), exhibited a maximum at 1% loading (Table 2) for both CNC and lignocellulose. The crystallite size of PVA, calculated using Equation (2), increased from 4.81 to 5.32 nm with CNCs, while it remained almost constant with lignocellulose (Figure S3). In similar systems, the crystal size of PVA is constant for loadings of 1–10% (around 9 nm) and decreases for loadings above 10% of other cellulose nanoparticles [37]. In the case of CNCs, at low loadings, the cellulose nanofibers remain well dispersed and serve effectively as nucleation centers. However, at higher loadings, their tendency to agglomerate increases, which limits dispersion and ultimately reduces the crystallinity index.

**Figure 6:**
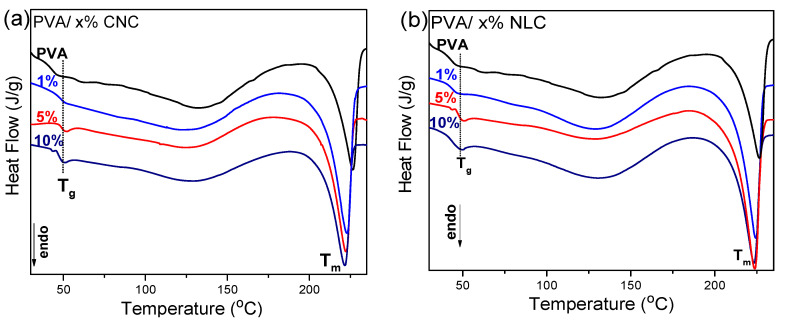
(**a**) DSC thermographs (first heat) of Pure PVA film and PVA composites with 1%, 5%, and 10% CNC. (**b**) DSC thermographs (first heat) of Pure PVA film and PVA composites with 1%, 5%, and 10% lignocellulose.

The Cr.I% of PVA was additionally calculated from DSC experiments, which also revealed the corresponding melting temperatures given in Table 2, as well as the glass transition temperature of the composites. The increase in the glass transition temperature (Tg) from 46 to ~50 °C upon incorporating 5–10% filler into the PVA matrix (Figure 6) is attributed to specific interactions of cellulosic fillers with the PVA matrix, primarily through hydrogen bonding (as discussed in the previous section), thus restricting the mobility of the polymer segments in accordance with references [38,39].

**Figure 7:**
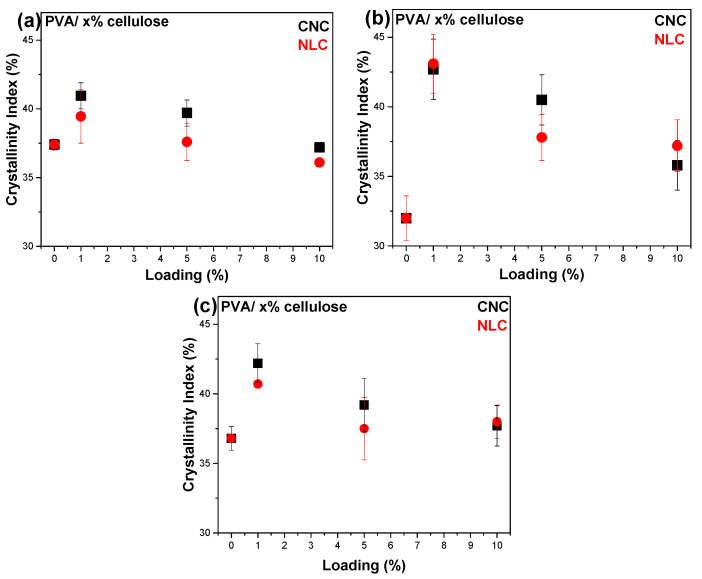
Comparative crystallinity index plots vs. inclusion loading, calculated by: (**a**) ATR/IR, (**b**) XRD, and (**c**) DSC. CNC composites (squares) and lignocellulose composites (circles).

The enhancement in crystallinity at 1% CNC loading can be attributed to the nucleating effect of the cellulose nanocrystals (Table 2). At this low concentration, the CNC particles were well-dispersed in the PVA matrix (Figure 2b) and acted, as already mentioned, as effective heterogeneous nucleation sites, facilitating the formation of more crystalline regions, which increased the crystallinity index from 36.8% to 42.2%. However, at higher loadings (5% and 10%), the PVA crystals were larger (Table 2), and more condensed CNCs may impede the alignment and mobility of the PVA chains, which diminishes their nucleating efficiency. As a result, the crystallinity index decreased to around 37%, similar to that of the pure PVA membrane [40]. Aggregate scatter plots of the calculated crystallinity index as a function of the inclusion percentage are shown in Figure 7 for DSC, XRD, and FTIR. The data points and error bars correspond to the average of three replicate samples for each technique.

#### 3.2.3. Anisotropy and Macromolecular Orientation

The mechanical properties of polymers and composites, along with their gas permeability properties, are strongly influenced by the induced anisotropy, which is a result of the preferential segmental orientation at the molecular level. Uniaxially or biaxially deformed polymers with a specific draw ratio (λ), defined as the length of the sample after deformation divided by its original length, are anisotropic materials with considerable mechanical alterations compared to isotropic polymers. There is a number of works that correlate the mechanical to the optical anisotropy characterized by spectroscopic techniques such as Raman, FTIR, or fluorescence. These techniques target the estimation of the segmental orientation distribution function, which evaluates sample anisotropy. The double-structure model divides semicrystalline polymers into two distinct regions: crystalline lamellae and amorphous areas. For the specific case of PVA-CNC composites, several works have indicated that the addition of CNC up to a certain wt.% results in increased orientation of the crystalline PVA fraction [41] or of the CNC particles [42] for a specific sample’s draw ratio. Further addition of the filler led to reduced orientation and consequently reduced mechanical properties. The latter was explained by the formation of agglomerates or a CNF network, which hindered alignment. Several parameters, such as PVA crystallinity, CNC size and aspect ratio, sample form (e.g., in fibers or films), and drawing conditions (draw ratio achieved, rate and temperature of drawing, etc.), are expected to play a key role in controlling the orientation. These parameters should be taken into account to optimize the material properties of interest. In order to verify the effect of sample deformation on the polymer orientation, Raman spectroscopy was applied to uniaxially cold-drawn PVA and its composite samples with CNC. The samples were submitted to draw ratios, λ = 2, 3; λ > 3 was not feasible since it is in the order of the experimental elongation at break (300%, see Table 3 below) for most of the samples. Selected vibrational features can, in principle, be used in order to quantify the segmental orientation of the crystalline and amorphous regions after collecting an appropriate number of polarized Raman spectra [41]. Alternatively, one can qualitatively characterize sample’s anisotropy using the polarization ratio, $R^{x}=\frac{I_{v-VV}^{x}}{I_{v-HH}^{x}}$, defined as the intensity of a selected vibrational band (x is the frequency of the band in cm^−1^) in a spectrum collected using polarization geometry of both incident and scattered radiation parallel to the stretching direction, divided by the corresponding intensity when using polarization geometry perpendicular to the stretching direction.

**Figure 8:**
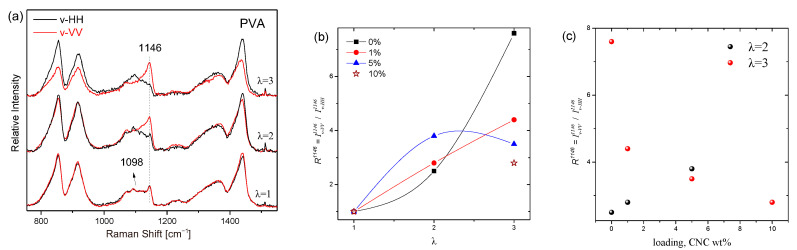
(**a**) Polarized Raman spectra at λ = 1, 2, and 3 of pure PVA, (**b**) orientation of the PVA crystal phase as a function of draw ratio for neat PVA and its composites, (**c**) orientation function as a function of loading for two distinct draw ratios.

The typical polarized Raman spectra of the isotropic and uniaxially stretched PVA films (λ = 2 and 3) are shown in Figure 8a. Most of the bands exhibit R < 1 values, indicative of vibrational modes possessing Raman tensors oriented in the vertical direction with respect to the polymer chain. For the band at 1146 cm^−1^, attributed to the PVA crystalline phase, R^1146^ > 1, with a tendency to increase at higher draw ratios. The calculated R^1146^ values for all samples as a function of λ are shown in Figure 8b. For neat PVA, the orientation increased with λ. For λ = 2, the addition of CNC enhanced the orientation of the PVA crystals with respect to the corresponding orientation of the neat PVA sample. This behavior appears to monotonically depend on the loading. Nevertheless, the best orientation of PVA crystals for λ = 3 was achieved for neat PVA samples and progressively decreased with the addition of the filler. The above-mentioned trends can be observed in Figure 8c, where the orientation of the PVA crystals is plotted as a function of the CNC loading. In summary, the addition of CNC at least up to 5 wt. % results in an increase of the orientation function for draw ratio 2 with respect to neat PVA. However, for λ > 2, the composites appeared to have an orientation lower than that of neat PVA. For 1 wt.% the orientation slightly increases, for 5 wt.% loading the orientation appears to reach a plateau, while for 10 wt.% the orientation is similar to that of neat PVA for λ = 2. The above experimental observations can be explained by the coexistence of a double network in the composite films, as proposed by Zhang et al. [43]. The crystallites (size 4.8 nm) formed a nanocrystal physical network between the amorphous regions of PVA and the additional physical network of the CNC filler (rod-like 50 nm × 400 nm), which also resided in the amorphous phase and interacted with the PVA chains through weaker hydrogen bonds. Uniaxial drawing of the samples primarily orients the larger CNC crystals, whose oriented network affects the orientation of the PVA crystallites. The mechanical properties of the stretched samples are expected to depend on the anisotropy of the samples for all components, i.e., CNC nanocrystals, PVA crystals, and amorphous regions. The orientation of the PVA nanocrystals could be the most critical of the three orientational parameters because it forms the most rigid of the two networks. However, a detailed and systematic study is required, which is beyond the scope of the current work, for higher loadings CNC both possible agglomerations or even a stronger coupling in the mobility of the coexisting networks hinder crystallite orientation (e.g., data points for 10% loading in Figure 8b,c) for samples under uniaxial drawing and cause no further increase in the mechanical properties (see Section 3.3.4).

### 3.3. Macroscopic Properties

#### 3.3.1. Swelling Properties

**Figure 9:**
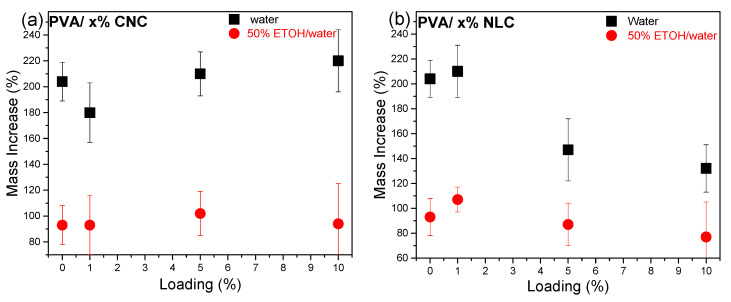
Mass increase plots of PVA membranes immersed in water (squares) and 50% ETOH/water (circles) for 48 h: (**a**) CNC composites and (**b**) lignocellulose composites.

In Figure 9a, the mass uptake scatter plot for water shows a minimum (20% lower value) at 1% CNC loading despite the hydrophilic nature of the inclusion, while higher loading gives values similar to those of the nominal PVA sample. The water mass uptake exhibited a reverse dependence on loading compared to the corresponding crystallinity dependence supported by all experimental techniques (DSC, XRD, and FTIR), as shown in Figure 7. Higher crystallinity results in a lower percentage and total volume of the amorphous phase, which is responsible for water uptake. According to Hossain et al. (2012), the three-dimensional network created by hydrogen bonding among the nanocrystals is believed to significantly affect the water absorption and swelling properties of the nanocomposites [44]. In contrast, hydrophobic lignocellulose (Figure 9b) affected the phenomenon at higher loadings by decreasing the uptake by up to 60%. In both composite systems, ethanol uptake was almost not detected, whereas in 50% ETOH/water (food simulant, according to EU regulations 10/2011, for plastic packaging in contact with food), all PVA membranes showed an uptake around 100% (close to the nominal), probably due to selective water absorption. It is interesting to note that the swelling properties of all samples are better for food packaging applications, referring to simulant D1 (50% ethanol). Their integrity was maintained after a long period of immersion in the simulant. Furthermore, a considerable decrease in swelling in both the D1 simulant and pure water was observed, especially for the composites containing high (>5%) NLC loadings, whose coherence was also found to be substantially improved. PVA is known to be selective toward ethanol in ethanol/water mixtures [45], which is explained by the almost separate dispersion of ethanol in the matrix with respect to water [46]. This behavior was found to be conserved in the cellulosic composites.

#### 3.3.2. Gas Permeability Measurements

Generally, neat PVA behaves as a barrier material, and its barrier properties benefit from its semicrystalline nature. Klepić et al. (2020) found that the CO_2_ permeability of neat PVA membranes with 75.3 μm thickness was 0.027 Barrer [47]. In the current work, the CO_2_ permeability of the PVA films (65 μm in thickness) was estimated to be 0.033 ± 0.009 Barrer, which is in remarkable agreement with the work of Klepić et al.

**Figure 10:**
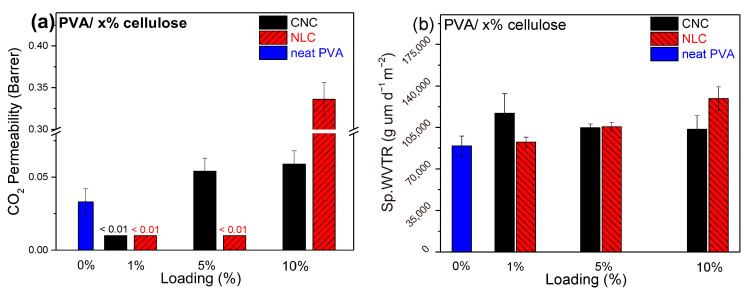
(**a**) CO_2_ permeability (Barrer) bar plot versus cellulose loading. The detection limit was 0.01 Barrer. (**b**) Sp.WVTR bar plot versus cellulose loading. CNC composites (black), lignocellulose composites (sparse red), and pure PVA (blue).

As depicted in Figure 10a, the composites with 1 wt.% loading of either CNC or LCN possess no measurable CO_2_ permeation. The same was observed for composites with 5 wt. % content in lignocellulose. The permeability of these samples was below the detection limit of mass spectrometry. This total barrier effect at 1% loading of both celluloses in CO_2_ can be attributed to the synergistic effect of increased crystallinity and good filler dispersion. At low loadings, such as 1 wt.%, the CNC or lignocellulose particles can efficiently act as nucleating agents, leading to a higher degree of crystallinity of the PVA matrix. Higher crystallinity usually creates denser regions with a consequent limitation of the corresponding amorphous regions, through which the passage of gas molecules is facilitated [48]. Thus, increased crystallinity should reduce the free volume of PVA, thereby hindering the CO_2_ diffusion.

In general, the incorporation of fillers can either enhance barrier properties by forming tortuous diffusion paths or, if poorly dispersed, create defects that may increase permeability [49]. Consequently, well-dispersed fillers at 1 wt.% loading must create a tortuous pathway for CO_2_ molecules, enhancing the barrier property. As the filler content increased, aggregation tended to occur, and the excessive presence of filler could disrupt the matrix, potentially forming microvoids or non-uniform regions. Therefore, the CO_2_ permeability of the PVA membrane with 10 wt.% CNC and lignocellulose were found to be 1.8 and 10.3 times higher than pure PVA, respectively. In conclusion, this interplay between crystallinity, dispersion, and overall morphology accounts for the best barrier effect at 1 wt.% loading, whereas, at higher loadings, the polymer matrix is disrupted, leading to CO_2_ permeability even higher than pure PVA. In addition, CNC loading fine-tuned the CO_2_ permeability by exhibiting a minimum at ~1 wt. %, while NLC exhibits a wider range of loading (1–5 wt.%) where the barrier properties are enhanced, but at even higher loading values, the barrier properties are found to strongly deteriorate. Taking into account the crystallinities of the samples containing the same wt. % loading on CNC or NLC, the barrier properties alteration cannot be explained solely by the PVA degree of crystallinity. The agglomeration of the filler could be a parameter affecting the permeability of the samples at higher wt.% loadings.

Finally, measurements of pure PVA on N_2_ and O_2_ permeability indicate a small permeation of both gases (0.0023 and 0.0013 Barrer, respectively), which is eliminated at any wt.% fraction.

#### 3.3.3. Water Vapor Transmittance Rate

Various factors may affect the WVTR of hydrophilic PVA membranes, such as the temperature and relative humidity of the experimental chamber, as long as the initial conditions of the samples remain firmly controlled [50]. In this context, in order to study the effect of different cellulosic inclusions (and their concentrations), a specific combination of RH and temperature was selected.

As shown in Figure 10b, the incorporation of cellulose-based fillers into the PVA matrix generally resulted in only a slight increase in the water vapor transmission rate (WVTR) compared to neat PVA. This behavior can be attributed to the hydrophilic nature of the fillers, which may introduce additional pathways for moisture transport through the membranes. At higher loadings, the effect was more pronounced in lignocellulose, likely due to the increased filler content disrupting the polymer network and providing more free volume for water vapor diffusion. In contrast, CNC shows a maximum at 1% loading, probably due to the well-dispersed nanocrystals that create a network of hydrophilic domains and channels that facilitate water vapor transport, even though the polymer’s crystallinity is enhanced.

#### 3.3.4. Mechanical Properties

**Figure 11:**
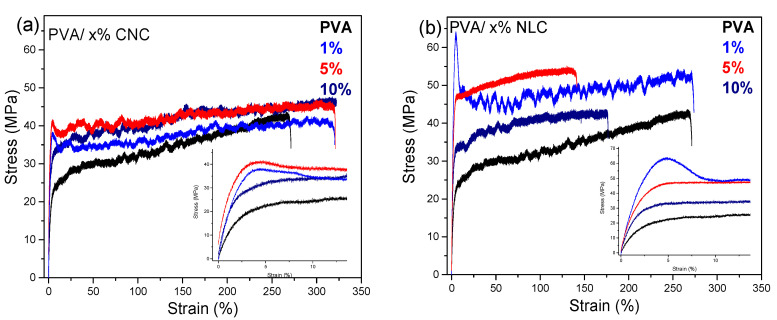
Stress−strain curves of PVA and PVA-CNC composites (**a**) and lignocellulose composites (**b**). Linear region (inlet).

Figure 11a illustrates the typical nonlinear stress−strain curves for the PVA and PVA/CNC composites. The response of hydrophilic membranes to stress is largely determined by the polymer network and its residual free and bound water. Upon applying a load, the polymer chains within the matrix initially reoriented, and only a small load was required to cause significant deformation. As the load further increased, the polymer chains gradually aligned uniformly, and the friction from both the polymer chains and the residual water led to a hardening effect, which in turn required higher stress to achieve additional deformation, resulting in a consecutive increase in the slope within the plastic deformation region (5–300% strain) [51,52].

Table 3 summarizes the tensile properties of the PVA composites with 1, 5, and 10% of either CNC or lignocellulose (the data appearing in the table are given as a bar plot in Figure S4). Compared to the neat matrix, all the reinforced samples exhibited enhanced Young’s modulus, tensile strength, and elongation at break. Notably, the PVA 1% CNC demonstrates the optimal mechanical performance by increasing 40% of Young’s modulus, 10% the tensile strength, and ~20% the elongation at break. Low loadings of needle-like cellulose inclusions (below 5%) provide the optimal combination of properties [13,16,51].

This reinforcing effect can be affected by factors such as the degree of crystallinity, crystal size, and intermolecular interactions of the matrix with the inclusions, as well as the morphology of the inclusions. Crystallinity is expected to influence the mechanical behavior of the composites and is likely one of the basic parameters for the ~50%/~170% increase in the Young’s modulus of the CNC/NLC composites. However, the relatively small variation in the estimated degree of crystallinity (5–10%) and the experimental finding that the ductility of at least the CNC composites is also improved suggest that an additional factor is the filler-matrix interactions, which also contributes as long as the dispersion quality of the filler is preserved. Finally, the ~170% increase in the Young’s modulus of the NLC composites should be attributed mainly to the fibrous structure of this particular filler. Well-dispersed CNCs help distribute stress more efficiently, allowing the polymer chains to elongate more before breaking, which is a consequence of the intermolecular hydrogen bond formation between PVA and CNC, as confirmed by FTIR spectroscopy. However, increasing the loading can restrict the mobility of the chains, making it more difficult for the polymer chains to deform under stress, leading to lower elongation at break, while the matrix becomes stiffer [25,53]. Furthermore, agglomerations of lignocellulose due to the long fibrous network lead to different behaviors in the mechanical properties of PVA composites. In Figure 11b, the previously referred strain-hardening effect is observed, but both the Young’s modulus and the elongation at break show differences with the inclusion type. While the stiffness of the composites is highly enhanced (up to 170% for 1–5% loading), the elongation at break is generally worse than that of the neat polymer. The increase in modulus is similar to that reported by Zimmermann et al. [54], where reinforced PVA with isolated cellulose fibrils resulted in a 250% increase in modulus at 10% loading. This behavior was observed at 1% loading in the present study, possibly due to randomly oriented lignocellulose fibrils. Moreover, the fibrous network restricts the PVA chain mobility at higher loadings due to its morphology and poor distribution. Only 1% loading has a similar value to PVA, giving the optimal concentration for a substantial increase in stiffness without affecting the ductility of the membrane. In both inclusion types, the 1 wt.% loading seems to be the optimal concentration for selectively enhancing the mechanical properties of PVA.

In summary, the swelling, gas permeation, and mechanical properties of the composites can be tailored based on the anticipated application as food packaging materials. Table 4 qualitatively summarizes the comparative properties of each composition investigated in the current work. The mechanical properties of PVA films and their crosslinked hydrogels strongly depend on their crystallinity [55]. However, cellulose-based composites exhibit diverse results in their mechanical properties, as stressed in a recent review by M.C. Alvarado ([56] and references therein). Notably, our results indicate that CNC improves Young’s modulus by ~50% and enhances the elongation at break by ~20%. These enhancements are attributed to the uniform dispersion of CNC, strong interfacial hydrogen bonding, and its role as a nucleating agent that boosts PVA crystallinity, as confirmed by DSC, XRD, and FTIR analyses. Higher Young’s moduli were measured for the NLC composites, which can be attributed to the increased crystallinity with respect to PVA, in addition to the morphology of the inclusion (which may explain the higher values compared to the CNC composites); however, this adversely affects the ductility at higher concentrations. Additionally, both CNC and NLC form complex, tortuous networks that significantly reduce gas permeability, with the most pronounced barrier effect observed at 1 wt.% loading for CNC and an interval between 1% and 5% for NLC. Higher filler loadings tend to cause agglomeration, which impacts both the mechanical (to a lesser extent in the loadings under investigation) and barrier performances. With respect to swelling properties, all samples exhibited reduced swelling when submerged in the D1 food simulant, while all cellulosic fillers only slightly affected water solubility, apart from NLC composites with >5% concentration. Furthermore, polarized Raman spectroscopy revealed an improvement in the PVA nanocrystal orientation at draw ratios of up to ~2 in composites with up to 5 wt.% CNC. However, at a draw ratio of ~3, the orientation appears to be less for all composites with respect to the neat polymer. This interesting result may be explained by the existence of a two-network model in the composites and should be considered in detail for applications that exploit stretch-induced anisotropy.

## 4. Conclusions

The incorporation of nanocellulose into PVA matrices is a promising strategy for creating sustainable, high-performance packaging materials. In this study, we systematically examined how CNC and NLC influence the physicochemical properties of PVA composites, thereby uncovering important structure-property relationships. Overall, our findings emphasize the need to carefully optimize the concentration of cellulosic inclusions in order to balance nucleation and dispersion within the polymer matrix and attain the desired properties of the packaging material. This study highlights the potential of nanocellulose as a critical additive in designing high-performance sustainable packaging materials and offers valuable insights into the structure-property interrelationships of PVA/cellulose composites. However, considering the main limitations identified, as well as the main advantages of incorporating cellulose nanocrystals into polyvinyl alcohol matrices for packaging materials, migration, and safety tests are the next steps required, especially for food packaging applications.

## Figures and Tables

**Table 1:** Samples and the number of replicates measured by each experimental technique.

Sample Description	Characterization Techniques								
	SEM	FTIR	XRD	DSC	Gas Perm.	WVTR	Swelling Prop.	Mechanical	Polarized Raman
PVA pure	1	5	3	3	3	2	3	8	2
PVA 1% CNC	1	5	3	3	3	2	3	8	2
PVA 5% CNC	1	5	3	3	3	2	3	8	2
PVA 10% CNC	1	5	3	3	3	2	3	8	2
PVA 1% NLC	1	5	3	3	3	2	3	8	2
PVA 5% NLC	1	5	3	3	3	2	3	8	2
PVA 10% NLC	1	5	3	3	3	2	3	8	2

**Table 2:** Crystallinity Index, melting temperature, and Crystal Size calculations of PVA and PVA composites.

Sample Description	Crystal Size (nm) XRD	Cr.I% XRD	Cr.I% ATR/FTIR	Cr.I% DSC	Tm (°C) DSC
PVA pure	4.81	32.0	37.4	36.8	226.0
PVA 1% CNC	4.76	42.7	40.9	42.2	222.6
PVA 5% CNC	5.13	40.5	39.7	39.2	221.9
PVA 10% CNC	5.32	35.8	37.2	37.7	221.3
PVA 1% NLC	4.65	43.1	39.5	40.7	223.6
PVA 5% NLC	4.80	37.8	37.6	37.5	222.8
PVA 10% NLC	4.77	37.2	36.1	38	222.3

**Table 3:** Young’s Modulus, Elongation at break, and Tensile Strength of PVA and PVA composite membranes.

Sample Description	Young Modulus (MPa)	Elongation at Break (%)	Tensile Strength at 100% Elongation (MPa)
PVA pure	1002 ± 86	270 ± 25	33 ± 2
PVA 1% CNC	1380 ± 130	330 ± 9	37 ± 4
PVA 5% CNC	1550 ± 130	320 ± 22	40 ± 4
PVA 10% CNC	1600 ± 98	300 ± 19	39 ± 5
PVA 1% NLC	2620 ± 170	270 ± 25	46 ± 3
PVA 5% NLC	2705 ± 170	127 ± 27	51 ± 3
PVA 10% NLC	1725 ± 130	170 ± 18	42 ± 2

**Table 4:** Comparative table of the macroscopic properties of the samples under investigation and the PVA nanocrystal orientation. Number of “●” symbols (one to five) indicates the quality factor of the corresponding macroscopic property).

Sample Description	Properties								
	WVTR	Barrier CO_2_	Swelling H_2_O	Swelling EtOH	Young Modulus	Elongation at Break	Tensile Strength	Best PVA Orientation	
								λ = 2	λ = 3
PVA pure	●●	●●●●	●●	●●●●●	●●	●●●●	●●	●	●●●●●
PVA 1% CNC	●	●●●●●	●●●	●●●●●	●●●	●●●●●	●●●	●●	●●●
PVA 5% CNC	●●	●●●	●●	●●●●●	●●●●	●●●●●	●●●●	●●●	●●
PVA 10% CNC	●●	●●●	●	●●●●●	●●●●	●●●●●	●●●●	-	●
PVA 1% NLC	●●	●●●●●	●●	●●●●	●●●●●	●●●●	●●●●	-	-
PVA 5% NLC	●●	●●●●●	●●●●	●●●●●	●●●●●	●●	●●●●●	-	-
PVA 10% NLC	●	●●	●●●●	●●●●●	●●●●	●●●	●●●●	-	-

## Data Availability

The original contributions presented in this study are included in the article/Supplementary Material. Further inquiries can be directed to the corresponding authors.

## Supplementary Materials

The following supporting information can be downloaded at: https://www.mdpi.com/article/10.3390/polym17141911/s1, Figure S1: Photographs of PVA cellulose composites exhibiting 1-5-10 wt.% of CNC (left) and lignocellulose (right); Figure S2: Deconvoluted XRD diffraction graph of PVA 5% CNC represented as method reference. Gaussian peak fitting is used for both crystalline (PVA and cellulose) and amorphous (PVA) depiction; Figure S3: Crystal size plots vs inclusion loading, calculated by XRD, using the Scherrer equation. CNC composites (square black) and lignocellulose composites (circle red); Figure S4. (a) Young Modulus plots vs loading. (b) Elongation at Break plots vs loading. (c) Tensile Strength at 100% vs loading. CNC composites (black), lignocellulose composites (red) and pure PVA (blue).

## Abbreviations

The following abbreviations are used in this manuscript:

wt.%	% weight percentage concentration
CNC	Cellulose Nano Crystals
NLC	Nano Lignocellulose
PVA	PolyVinyl Alcohol
ATR-FTIR	Attenuated total reflectance- Fourier transform infrared spectroscopy
XRD	X-Ray Diffraction
DSC	Differential Scanning Calorimetry
WVTR	Water Vapor Transmittance Rate
Cr.I%	% Crystallinity Index

## References

[1] Siracusa V., Rocculi P., Romani S., Rosa M.D. (2008). Biodegradable polymers for food packaging: A review. Trends Food Sci. Technol..

[2] Rhim J.-W., Park H.-M., Ha C.-S. (2013). Bio-nanocomposites for food packaging applications. Prog. Polym. Sci..

[3] Ben Halima N. (2016). Poly(vinyl alcohol): Review of its promising applications and insights into biodegradation. RSC Adv..

[4] Muhammad F.F., Aziz S.B., Hussein S.A. (2015). Effect of the dopant salt on the optical parameters of PVA:NaNO_3_ solid polymer electrolyte. J. Mater. Sci. Mater. Electron..

[5] Mansur H.S., Sadahira C.M., Souza A.N., Mansur A.A. (2008). FTIR spectroscopy characterization of poly (vinyl alcohol) hydrogel with different hydrolysis degree and chemically crosslinked with glutaraldehyde. Mater. Sci. Eng. C.

[6] Hassan C.M., Peppas N.A. (2000). Structure and Applications of Poly(vinyl alcohol) Hydrogels Produced by Conventional Crosslinking or by Freezing/Thawing Methods. Biopolymers PVA Hydrogels, Anionic Polymerisation Nanocomposites.

[7] Oun A.A., Shin G.H., Rhim J.-W., Kim J.T. (2022). Recent advances in polyvinyl alcohol-based composite films and their applications in food packaging. Food Packag. Shelf Life.

[8] Aslam M., Kalyar M.A., Ali Raza Z. (2018). Polyvinyl alcohol: A review of research status and use of polyvinyl alcohol based nanocomposites. Polym. Eng. Sci..

[9] Faruk O., Bledzki A.K., Fink H.-P., Sain M. (2012). Biocomposites reinforced with natural fibers: 2000–2010. Prog. Polym. Sci..

[10] Spitalsky Z., Tasi D., Papgelis K., Galioti C. (2010). Carbon nanotube-polymer composites: Chemistry, processing mechanical and electrical properties. Prog. Polym. Sci..

[11] Wang F., Hu Z., Ouyang S., Wang S., Liu Y., Li M., Wu Y., Li Z., Qian J., Wu Z. (2024). Application progress of nanocellulose in food packaging: A review. Int. J. Biol. Macromol..

[12] Fortunati E., Puglia D., Monti M., Santulli C., Maniruzzaman M., Kenny J.M. (2013). Cellulose nanocrystals extracted from okra fibers in PVA nanocomposites. J. Appl. Polym. Sci..

[13] Jahan Z., Niazi M.B.K., Gregersen Ø.W. (2018). Mechanical, thermal and swelling properties of cellulose nanocrystals/PVA nanocomposites membranes. J. Ind. Eng. Chem..

[14] Popescu M.-C. (2017). Structure and sorption properties of CNC reinforced PVA films. Int. J. Biol. Macromol..

[15] Cheng Q., Wang S., Rials T.G. (2009). Poly(vinyl alcohol) nanocomposites reinforced with cellulose fibrils isolated by high intensity ultrasonication. Compos. Part A: Appl. Sci. Manuf..

[16] Liu D., Sun X., Tian H., Maiti S., Ma Z. (2013). Effects of cellulose nanofibrils on the structure and properties on PVA nanocomposites. Cellulose.

[17] Fortunati E., Puglia D., Luzi F., Santulli C., Kenny J., Torre L. (2013). Binary PVA bio-nanocomposites containing cellulose nanocrystals extracted from different natural sources: Part I. Carbohydr. Polym..

[18] Du J., Guo J., Zhu Q., Guo J., Gu J., Wu Y., Ren L., Yang S., Jiang J. (2025). Enhancement of Polyvinyl Alcohol-Based Films by Chemically Modified Lignocellulosic Nanofibers Derived from Bamboo Shoot Shells. Polymers.

[19] Zhang S., Wang Z., Li C., Zheng Y., Xu J. (2025). A strong, excellent water resistance, and anti-ultraviolet poly(vinyl alcohol)/lignocellulose/poly(butylene adipate-co-terephthalate) composite with “sandwich” structure. Int. J. Biol. Macromol..

[20] Lv C., Liu D., Tian H., Xiang A. (2020). Non-isothermal crystallization kinetics of polyvinyl alcohol plasticized with glycerol and pentaerythritol. J. Polym. Res..

[21] (1995). Standard Test Methods for Water Vapor Transmission of Materials.

[22] Bounos G., Andrikopoulos K., Moschopoulou H., Lainioti G., Roilo D., Checchetto R., Ioannides T., Kallitsis J., Voyiatzis G. (2017). Enhancing water vapor permeability in mixed matrix polypropylene membranes through carbon nanotubes dispersion. J. Membr. Sci..

[23] Kumaran M. (1998). Interlaboratory Comparison of the ASTM Standard Test Methods for Water Vapor Transmission of Materials (E 96-95). J. Test. Eval..

[24] (2018). Standard Test Method for Tensile Properties of Thin Plastic Sheeting.

[25] Roohani M., Habibi Y., Belgacem N.M., Ebrahim G., Karimi A.N., Dufresne A. (2008). Cellulose whiskers reinforced polyvinyl alcohol copolymers nanocomposites. Eur. Polym. J..

[26] Tretinnikov O.N., Zagorskaya S.A. (2012). Determination of the degree of crystallinity of poly(vinyl alcohol) by FTIR spectroscopy. J. Appl. Spectrosc..

[27] Maréchal Y., Chanzy H. (2000). The hydrogen bond network in I_β_ cellulose as observed by infrared spectrometry. J. Mol. Struct..

[28] Gohil J.M., Bhattacharya A., Ray P. (2006). Studies on the Crosslinking of Poly (Vinyl Alcohol). J. Polym. Res..

[29] Socrates G. (2001). Infrared and Raman Characteristic Group Frequencies.

[30] Larkin P.J. (2011). Infrared and Raman Spectroscopy Principles and Spectral Interpretation.

[31] Oh S.Y., Yoo D.I., Shin Y., Kim H.C., Kim H.Y., Chung Y.S., Park W.H., Youk J.H. (2005). Crystalline structure analysis of cellulose treated with sodium hydroxide and carbon dioxide by means of X-ray diffraction and FTIR spectroscopy. Carbohydr. Res..

[32] French A.D. (2014). Idealized powder diffraction patterns for cellulose polymorphs. Cellulose.

[33] Zhang W., He X., Li C., Zhang X., Lu C., Zhang X., Deng Y. (2014). High performance poly (vinyl alcohol)/cellulose nanocrystals nanocomposites manufactured by injection molding. Cellulose.

[34] Ricciardi R., Auriemma F., De Rosa C., Lauprêtre F. (2004). X-ray Diffraction Analysis of Poly(vinyl alcohol) Hydrogels, Obtained by Freezing and Thawing Techniques. Macromolecules.

[35] Mallapragada S.K., Peppas N.A. (1996). Dissolution mechanism of semicrystalline poly(vinyl alcohol) in water. J. Polym. Sci. Part B Polym. Phys..

[36] Karimi K., Taherzadeh M.J. (2016). A critical review of analytical methods in pretreatment of lignocelluloses: Composition, imaging, and crystallinity. Bioresour. Technol..

[37] Voronova M.I., Surov O.V., Guseinov S.S., Barannikov V.P., Zakharov A.G. (2015). Thermal stability of polyvinyl alcohol/nanocrystalline cellulose composites. Carbohydr. Polym..

[38] Bernhard W. (2005). Thermal Analysis of Polymeric Materials.

[39] Menczel J.D., Prime R.B. (2008). Thermal Analysis of Polymers: Fundamentals and Applications.

[40] Ning N., Fu S., Zhang W., Chen F., Wang K., Deng H., Zhang Q., Fu Q. (2012). Realizing the enhancement of interfacial interaction in semicrystalline polymer/filler composites via interfacial crystallization. Prog. Polym. Sci..

[41] Peng J., Ellingham T., Sabo R., Turng L.-S., Clemons C.M. (2014). Short cellulose nanofibrils as reinforcement in polyvinyl alcohol fiber. Cellulose.

[42] Shrestha S., Montes F., Schueneman G.T., Snyder J.F., Youngblood J.P. (2018). Effects of aspect ratio and crystal orientation of cellulose nanocrystals on properties of poly(vinyl alcohol) composite fibers. Compos. Sci. Technol..

[43] Zhang N., Pang Y., Li Z., Yang C., Zong L., Yang H., Wu H., Duan Y., Zhang J. (2023). Rubber-like and biodegradable poly (vinyl alcohol) composites with triple networks for high-efficiency solvent barrier. Compos. Sci. Technol..

[44] Hossain K.M.Z., Ahmed I., Parsons A.J., Scotchford C.A., Walker G.S., Thielemans W., Rudd C.D. (2012). Physico-chemical and mechanical properties of nanocomposites prepared using cellulose nanowhiskers and poly(lactic acid). J. Mater. Sci..

[45] Farid O., Mansour F., Habib M., Robinson J., Tarleton S. (2016). Investigating the sorption influence of poly(vinyl alcohol) (PVA) at different crosslinking content. J. Environ. Chem. Eng..

[46] Zhang Q.G., Liu Q.L., Chen Y., Wu J.Y., Zhu A.M. (2009). Microstructure dependent diffusion of water–ethanol in swollen poly(vinyl alcohol): A molecular dynamics simulation study. Chem. Eng. Sci..

[47] Klepić M., Setničková K., Lanč M., Žák M., Izák P., Dendisová M., Fuoco A., Jansen J.C., Friess K. (2020). Permeation and sorption properties of CO_2_-selective blend membranes based on polyvinyl alcohol (PVA) and 1-ethyl-3-methylimidazolium dicyanamide ([EMIM][DCA]) ionic liquid for effective CO_2_/H_2_ separation. J. Membr. Sci..

[48] Idris A., Muntean A., Mesic B., Lestelius M., Javed A. (2021). Oxygen Barrier Performance of Poly(vinyl alcohol) Coating Films with Different Induced Crystallinity and Model Predictions. Coatings.

[49] Idris A., Muntean A., Mesic B. (2022). A review on predictive tortuosity models for composite films in gas barrier applications. J. Coat. Technol. Res..

[50] Abdullah Z.W., Dong Y., Han N., Liu S. (2019). Water and gas barrier properties of polyvinyl alcohol (PVA)/starch (ST)/glycerol (GL)/halloysite nanotube (HNT) bionanocomposite films: Experimental characterisation and modelling approach. Compos. Part B Eng..

[51] Gonzalez J.S., Ludueña L.N., Ponce A., Alvarez V.A. (2014). Poly(vinyl alcohol)/cellulose nanowhiskers nanocomposite hydrogels for potential wound dressings. Mater. Sci. Eng. C Mater. Biol. Appl..

[52] Ma R., Xiong D., Miao F., Zhang J., Peng Y. (2009). Novel PVP/PVA hydrogels for articular cartilage replacement. Mater. Sci. Eng. C.

[53] Millon L.E., Oates C.J., Wan W. (2009). Compression properties of polyvinyl alcohol-bacterial cellulose nanocomposite. J. Biomed. Mater. Res. Part B Appl. Biomater..

[54] Zimmermann T., Pöhler E., Geiger T. (2004). Cellulose Fibrils for Polymer Reinforcement. Adv. Eng. Mater..

[55] Lin S., Liu X., Liu J., Yuk H., Loh H.-C., Parada G.A., Settens C., Song J., Masic A., McKinley G.H. (2019). Anti-fatigue-fracture hydrogels. Sci. Adv..

[56] Alvarado M.C. (2024). Recent progress in polyvinyl alcohol (PVA)/nanocellulose composite films for packaging applications: A comprehensive review of the impact on physico-mechanical properties. Food Bioeng..

