# Supplementary material for: Structure-Properties Correlations of PVA-Cellulose Based Nanocomposite Films for Food Packaging Applications
Source: Polymers (Basel). 2025 Jul 10;17(14):1911. doi: 10.3390/polym17141911 (PMC12299579; doi:10.3390/polym17141911)
Supplement: Supplementary file 1 [file polymers-17-01911-s001.zip › polymers-3722289-supplementary.docx]

***Supplementary Information***

**Structure-properties correlations of PVA-cellulose based nanocomposite films for food packaging applications**

*Konstantinos Papapetros^1,2^, Georgios N. Mathioudakis^1^, Dionysios Vroulias ^1^, Nikolaos Koutroumanis^3^, George A. Voyiatzis^1*^, Konstantinos S. Andrikopoulos^1,4*^*

*^1^Foundation for Research and Technology – Hellas (FORTH), Institute of Chemical Engineering Science (ICE-HT), Stadiou St., P.O. Box 1414, GR 265 04 Rio-Patras Greece*

*^2^Department of Chemical Engineering, University of Patras, Patras, GR-265 04, Greece*

*^3^*Application Driven Research & Innovative Engineering (ADRINE), Patras Science Park, Stadiou Street, Platani, Patras, 26504 Greece

*^4^Department of Physics, University of Patras, GR-26504, Patras, Greece*

**Correspondence: gvog@iceht.forth.gr, kandriko@upatras.gr*

| ***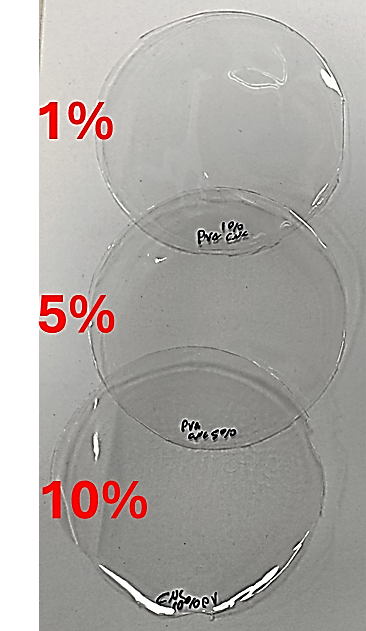*** | ***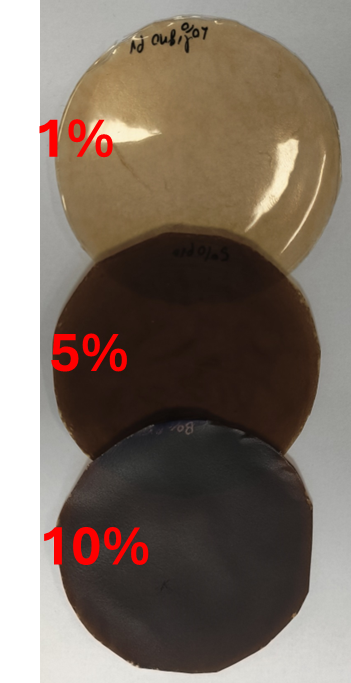*** |
| --- | --- |
| ***Figure S1:*** *Photographs of* *PVA cellulose composites exhibiting 1-5-10 wt.% of CNC (left) and lignocellulose (right).* | |

| **** |
| --- |
| ***Figure S2:*** *Deconvoluted* *XRD diffraction graph of PVA 5% CNC represented as method reference. Gaussian peak fitting is used for both crystalline (PVA and cellulose) and amorphous (PVA) depiction.* |

| **** |
| --- |
| ***Figure S3:*** *Crystal size plots vs inclusion loading, calculated by XRD, using the Scherrer equation. CNC composites (square black) and lignocellulose composites (circle red).* |

| **a)** | **b)** |
| --- | --- |
| **c)** | |
| ***Figure S4.*** *(a) Young Modulus plots vs loading. (b) Elongation at Break plots vs loading. c) Tensile Strength at 100% vs loading. CNC composites (black), lignocellulose composites (red) and pure PVA (blue).* | |
